# Intra-Species Interactions in *Streptococcus pneumoniae* Biofilms

**DOI:** 10.3389/fcimb.2021.803286

**Published:** 2022-01-05

**Authors:** Carina Valente, Ana R. Cruz, Adriano O. Henriques, Raquel Sá-Leão

**Affiliations:** ^1^ Laboratory of Molecular Microbiology of Human Pathogens, Instituto de Tecnologia Química e Biológica António Xavier, Universidade Nova de Lisboa, Oeiras, Portugal; ^2^ Laboratory of Microbial Development, Instituto de Tecnologia Química e Biológica António Xavier, Oeiras, Portugal

**Keywords:** biofilms, co-colonization, colonization, multiple carriage, competition, intraspecies interactions, *Streptococcus pneumoniae*

## Abstract

*Streptococcus pneumoniae* is a human pathogen responsible for high morbidity and mortality worldwide. Disease is incidental and is preceded by asymptomatic nasopharyngeal colonization in the form of biofilms. Simultaneous colonization by multiple pneumococcal strains is frequent but remains poorly characterized. Previous studies, using mostly laboratory strains, showed that pneumococcal strains can reciprocally affect each other’s colonization ability. Here, we aimed at developing a strategy to investigate pneumococcal intra-species interactions occurring in biofilms. A 72h abiotic biofilm model mimicking long-term colonization was applied to study eight pneumococcal strains encompassing 6 capsular types and 7 multilocus sequence types. Strains were labeled with GFP or RFP, generating two fluorescent variants for each. Intra-species interactions were evaluated in dual-strain biofilms (1:1 ratio) using flow cytometry. Confocal microscopy was used to image representative biofilms. Twenty-eight dual-strain combinations were tested. Interactions of commensalism, competition, amensalism and neutralism were identified. The outcome of an interaction was independent of the capsular and sequence type of the strains involved. Confocal imaging of biofilms confirmed the positive, negative and neutral effects that pneumococci can exert on each other. In conclusion, we developed an experimental approach that successfully discriminates pneumococcal strains growing in mixed biofilms, which enables the identification of intra-species interactions. Several types of interactions occur among pneumococci. These observations are a starting point to study the mechanisms underlying those interactions.

## Introduction

*Streptococcus pneumoniae* is an important bacterial pathogen associated with high morbidity and mortality worldwide (GBD, 2018). Nevertheless, disease is a rare event compared with the frequency of asymptomatic nasopharyngeal colonization (Weiser et al., 2018).

Nasopharyngeal colonization is particularly frequent in young children (Gray et al., 1980), where multiple pneumococcal strains can simultaneously colonize the same host, a phenomenon known as multiple serotype carriage or co-colonization (Turner et al., 2011; Valente et al., 2012; Wyllie et al., 2014; Kamng’ona et al., 2015; Kandasamy et al., 2015; Valente et al., 2016b). Co-colonization is frequent, reaching up to 40% or 50%, depending on the geographical setting and the methodology used for its detection (Turner et al., 2011; Valente et al., 2012; Wyllie et al., 2014; Kamng’ona et al., 2015; Kandasamy et al., 2015; Valente et al., 2016b).

During colonization, pneumococci form biofilms that confer protection from the host and enable horizontal gene transfer, the main evolutionary mechanism of this species (Barnes et al., 1995; Hiller et al., 2010; Wei and Havarstein, 2012).

Several lines of evidence indicate that intra-species competition (*i.e.*, competition between pneumococcal strains) occurs during co-colonization and has a determinant role in shaping its epidemiology and population structure. For example, the dominance of a limited subset of capsular types (typically 5-6 types) in carriage, when several are known to circulate in a population, is a clear indication that strains have different competitive abilities to colonize (Hausdorff et al., 2005). Introduction of pneumococcal conjugate vaccines worldwide also indicates that, following serotype replacement, the population structure of pneumococci is reshaped due to expansion of a limited number of non-vaccine types in carriage and disease (Moore et al., 2015; Waight et al., 2015; Azarian et al., 2020). In addition, using a mouse model of colonization, Lipsitch and co-workers showed that the presence of a resident pneumococcal strain could affect colonization by a second (challenger) strain (Lipsitch et al., 2000).

The mechanisms mediating pneumococcal intra-species competition have been addressed in a few subsequent studies. Trzciński et al. tested clinical isolates and isogenic variants of six capsular types in a mouse model of multiple-serotype carriage and observed that the competitive ability of a strain to colonize depends strongly, but not exclusively, on the composition of its polysaccharide capsule (Trzciński et al., 2015). Apart from the capsule, the *blp* (bacteriocin-like peptide) locus and the competence system were also implicated in competitive interactions. Dawid et al., used a 19A clinical isolate and its *blp* bacteriocin deleted derivative, to demonstrate that the *blp* locus promotes intra-species competition, both *in vitro* (using overlay assays) and *in vivo* (using a murine model of colonization) (Dawid et al., 2007). In a follow-up study, Wholey et al. observed a cross-stimulation between the *blp* and competence systems with implications for intra-species interactions (Wholey et al., 2016). Moreover, in dual-strain biofilms, bacteriocin producing pneumococci, not only had a competitive advantage over non-producers, but also increased DNA exchange through natural transformation (Wholey et al., 2016). Using an infant mouse model of colonization, Shen et al. showed that the competitive advantage of a resident strain is quorum sensing-dependent and is mediated by the late-competence activated fratricide effectors CbpD and CibAB (Shen et al., 2019). Finally, Wu et al. tested three clinical isolates, of serotypes 6B, 19F and 23F, in an eight-hour dual-strain biofilm model and showed that strain 19F was outcompeted by strains 6B and 23F through a contact-dependent unknown mechanism (Wu et al., 2017).

While other types of interactions, beyond competition, are theoretically possible among pneumococci, they have remained mostly undescribed. One exception are the studies of Trzciński et al. and Wu et al., who described strain combinations where co-existence was observed suggesting a neutral interaction (Trzciński et al., 2015; Wu et al., 2017). In addition, while using isogenic laboratory strains (D39 wt and D39 *pspA/pspC* null mutant) to study natural transformation in multi-strain biofilms, Marks and co-authors found that mixed populations of pneumococci may increase the adaptive potential of less fit strains (Marks et al., 2012), suggesting that commensalism may occur between pneumococci.

Despite significant advances on the study of pneumococcal co-colonization during the last two decades, progress has been hampered by methodological constraints associated with the need to quantitatively distinguish cells of different strains when in mixed-strain experimental conditions. While it is recognized that there is significant within strain genetic diversity in pneumococci, the number of strains being used in such studies has been limited and has mainly relied on the use of laboratory strains and its derivatives.

In this study, we developed a strategy to study pneumococci in multi-strain biofilms and applied it to a diverse set of strains which included natural isolates obtained from samples collected from colonized human subjects. We show that various types of intra-species interactions, including commensalism, do occur and can be quantitatively characterized.

## Materials and Methods

### Study Collection

The study collection consisted of 12 strains previously isolated from nasopharyngeal samples of healthy children in which co-colonization was detected (Sá-Leão et al., 2009; Valente et al., 2012; Nunes et al., 2016; Valente et al., 2016a; Valente et al., 2016b; Félix et al., 2021) and two laboratory strains, D39 and TIGR4, commonly used in pneumococcal studies worldwide. Epidemiological relevance of selected strains and their serotypes and genotypes are indicated in **Tables 1**, **2**. Four strains were non-encapsulated and were designated as non-typable (NT).

**Table 1:** Rationale for selection of *S. pneumoniae* strains based on epidemiological relevance.

Serotype	PCV13 serotype	Observations	References
3	Yes	High invasive disease potential Increasing in PCV13 era	(Sá-Leão et al., 2011; Horácio et al., 2016)
15A	No	Increasing in carriage in PCV13 era	(Félix et al., 2021)
19A	Yes	Highly prevalent in carriage and disease before the introduction of PCV13	(Isturiz et al., 2017)
19F	Yes	Remains in carriage in PCV13 era	(Nunes et al., 2016; Félix et al., 2021)
22F; 33F	No	Increasing in carriage in PCV13 era Important cause of invasive pneumococcal disease Targeted by upcoming PCV15 and PCV20 vaccines	(Moore et al., 2015; Félix et al., 2021)
NT	No	Non-encapsulated Very frequent in colonization Frequently detected in co-colonization Associated with multidrug resistance Associated to conjunctivitis outbreaks	(Marks et al., 2012; Valentino et al., 2014; Valente et al., 2016b)

PCV13, PCV15 and PCV20 correspond to 13-valent, 15-valent and 20-valent pneumococcal conjugate vaccines. NT - non-typeable.

**Table 2:** Bacterial strains initially chosen for the study of intra-species interactions.

Strain	Serotype	MLST^1^	Observation^2^	Reference
D39	2	595	selected	(Avery et al., 1944)
TIGR4	4	205	selected	(Tettelin et al., 2001)
2099	3	260	–	(Nunes et al., 2016)
7632	15A	8322	selected	(Nunes et al., 2016)
5262.1	19A	276	–	(Sá-Leão et al., 2009)
5902	19A	63	–	(Simões et al., 2011)
1990	19F	177	selected	(Nunes et al., 2016)
5756	22F	433	selected	(Sá-Leão et al., 2009)
8046	22F	9161	–	(Nunes et al., 2016)
8276	33F	717	–	(Félix et al., 2021)
5262.2	NT	344	–	(Sá-Leão et al., 2009)
5435	NT	344	selected	(Sá-Leão et al., 2009)
7031	NT	344	selected	(Nunes et al., 2016)
6209	NT	4583	selected	(Simões et al., 2011)

MLST, multilocus sequence typing; NT, non-typeable (non-encapsulated). ^1^STs mainly associated with colonization isolates (www.pubmlst.org).

^2^Strains tested in dual-strain biofilms are indicated as “selected”. Other stains did not meet one or more validation criteria and were excluded.

### Fluorescent Labelling of *S. pneumoniae* Strains

Each strain was labelled with GFP or RFP generating two fluorescently labelled strains. Constructs P*_hlpA_*-*hlpA*-*gfp*_Cam^r^ (for GFP-labelling) and P*_hlpA_*-*hlpA_hlpA*-*rfp*_Cam^r^ (for RFP-labelling), kindly provided by Jan-Willem Veening, were used (Kjos et al., 2015). These constructs bear a translational fusion of the histone-like protein A gene (*hlpA*) to GFP or RFP, where the fusion proteins are produced under the control of the *hlpA* promoter; the constructs also carry a transcriptional fusion to a chloramphenicol resistance gene. Constructs were first integrated by allelic replacement in the genome of *S. pneumoniae* D39 (laboratory strain) generating strains D39_GFP and D39_RFP. DNA was extracted from either D39_GFP or D39_RFP and amplified by PCR using primers previously described (Kjos et al., 2015). PCR products were purified with Exo-SAP and used for transformation of other strains.

Two transformation protocols, in liquid medium and in biofilms, were used. For transformation in liquid medium cells were grown in C+Y (pH 7.4) (Junges et al., 2017) to an optical density of 0.5 at 600nm (OD_600_), diluted at a 1:100 ratio and grown until an OD_600nm_ of 0.1 was reached; at this time, 100ng/mL of CSP1 (NH2-EMRLSKFFRDFILQRKK-COOH) or CSP2 (NH2-EMRISRIILDFLFLRKK-COOH) (Mimotopes, Australia) and 400ng/mL of DNA were added. The mixture was incubated for 3h at 37°C.

For clinical isolates which did not yield transformants using the conditions described above, transformation in biofilms was attempted. Cells were grown in C+Y (pH 7.4) (Junges et al., 2017) to an OD_600_ of 0.5, diluted at a 1:100 ratio, grown until an OD_600_ of 0.2 and transferred to 24-well plates (Costar, Corning), in a final volume of 2mL per well. Plates were incubated for 4h at 34°C in a 5% CO_2_ atmosphere to promote biofilm formation (Wei and Havarstein, 2012). The supernatant was carefully aspirated to remove planktonic bacteria and replaced with 2mL of fresh medium supplemented with 0.5µg/mL of DNA and the appropriate CSP at a final concentration of either 100ng/mL or 1000ng/mL.

In both protocols transformants were selected on TSA blood plates supplemented with 4µg/mL chloramphenicol. For selected clones, the presence of the fusion constructs at the *hlpA* locus was first verified by PCR.

### Fluorescence Microscopy

For selected clones resulting from the integration of the P*_hlpA_*-*hlpA*-*gfp* or P*_hlpA_*-*hlpA_hlpA*-*rfp* fusions, production of GFP or RFP was assessed by fluorescence microscopy. Two mL of exponential phase culture were centrifuged at 5900g for 10min and pellets were resuspended in PBS. Cell suspensions were mounted on a slide covered with a thin layer of 1.7% agarose. Images were acquired on a Leica DM 6000B fluorescence microscope controlled by MetaMorph V5.8 software through a 100X 1.4 NA oil immersion objective and captured with an Andor iXon 885 EMCCD camera. Filters for image acquisition were set for contrast phase optics, TX2 (excitation: 560/40nm; emission: 645/75nm), and FITC (excitation: 480/40nm; emission: 527/30nm). Exposure times were 50ms for contrast phase, 3000ms for TX2, and 500ms for FITC.

### Biofilm Growth Medium for the Study of Intra-Species Interactions

Strains were grown in a modified version of the chemically defined medium described by van de Rijn and Kessler, herein named mCDM, in which the final concentration of cysteine-HCl was increased to 0.8mg/mL (instead of 0.5mg/mL), choline chloride was added at a final concentration of 1mg/mL, except when otherwise indicated. The carbon source was galactose (10mg/mL) instead of glucose (the complete recipe of mCDM is given in **Table S1**). Cysteine promotes faster bacterial growth as it can act as a radical scavenger and as an additional amino-acid source. Choline is a nutritional requirement for pneumococcal growth and was added at a concentration of 1mg/mL to promote better growth (Rane and Subbarow, 1940; Tomasz, 1967). Galactose was chosen as it is the most abundant sugar in the human nasopharynx (Blanchette et al., 2016). All experiments were carried out in 24-well plates (Costar, Corning) and the cultures incubated at 34°C (Keck et al., 2000) in a 5% CO_2_-enriched atmosphere.

### *In Vitro* Biofilm Model

An in-house developed abiotic biofilm model mimicking long term-colonization was used. Strains were first grown in mCDM in planktonic growth conditions until mid-exponential phase. A 1:500 dilution of each strain of interest, corresponding to 10^5^ cells in 2.5mL of mCDM, was transferred to 24-well plates and incubated at 34°C in 5% CO_2_ for 72 hours. At 24h and 48h, 2.0mL of spent medium were carefully removed and 2.0mL of pre-warmed fresh medium was added avoiding disturbance of the biofilm attached to the bottom of the well. At 72h, biofilms were resuspended in 300µL of PBS and either serially diluted for colony forming units (CFU) counts or sonicated to separate aggregates prior to flow cytometry analysis (described below). Sonication was done in a Bioruptor UCD-300 apparatus with an ultrasonic wave frequency of 20KHz (12 cycles of 10s at high intensity with 30s break between pulses).

### Single-Strain and Dual-Strain Biofilm

To evaluate interactions between strains, dual-strain biofilms were grown in a 1:1 ratio in the conditions described above. To allow for strain discrimination, dual-strain biofilm experiments used one GFP-labelled strain and one RFP-labelled strain. As a control, each pair of strains was also tested with the reverse fluorescent markers. For comparative analysis, single-strain biofilms of the same strain variants were obtained in parallel. For all experiments three biological replicates were done and each included three technical replicates.

### Flow Cytometry for Assessment of Cell Counts and Cell Density in Single-Strain and Dual-Strain Biofilm

Cell counts of single- and dual-strain biofilms were obtained by flow cytometry as a proxy for cell viability. Biofilms disrupted by sonication (see above) were diluted in an appropriate volume of PBS to achieve a rate of 500 events/s. Cell counts were obtained in a Bio-Rad S3e Cell Sorter controlled by the ProSort software v1.6 using the following parameters: nozzle size of 100microns, lasers of 488nm and 561nm, and filters FL1 (525/30nm) and FL3 (615/25nm) for data collection of GFP- and RFP-labelled cells, respectively. Flow cytometry conditions were set as: FSC 311V, SSC 240V, FL1 811V and FL3 335V. As the detection limit of the Bio-Rad S3e Cell Sorter is above the expression levels of RFP-labelled cells, these were detected as non-labelled in the FL1 filter.

For each pair of strains tested in dual-strain biofilm, the corresponding single-strain biofilms were first analyzed. Cells in single biofilms were counted (about 30000 events). Cells of the RFP-labelled strain were used to establish a “red region” in the FL1 Area log plot. Similarly, cells of the GFP-labelled strain were used to establish a “green region” in the same FL1 Area log plot. Potential overlap between these two regions was investigated and, when occurring, the relative proportion of GFP-labelled cells present in the “red region” was determined. For dual-strain biofilms, the relative proportion of the RFP-labelled and GFP-labelled strains was estimated by counting 30000 events and determining how many occurred in the red or green regions. When appropriate, a correction (based on the single-strain biofilm results) was applied. Finally, to estimate the cell density for each strain in single and dual-strain biofilms, total cell counts in 50µL were obtained.

### Identification of Intra-Species Interactions

To investigate whether intra-species interactions occurred in the dual-strain biofilm experiments results obtained for a given pair of strains were combined in order to increase statistical power. For each strain, total cell counts obtained in single-strain biofilms (GFP- plus RFP-labelled variant cells) were compared to the total cell counts obtained for that strain in dual-strain biofilms. Interactions were considered to occur when statistically significant differences were observed (p<0.05). Upon detection of an interaction, a ratio between the geometric means of cell counts in dual- and single-strain biofilms was calculated. Negative interactions were defined by ratios <1 and positive interactions were defined by ratios >1.

### Confocal Microscopy for Biofilm Imaging

Biofilms were grown in 24-well microscope slides suitable for confocal microscopy (IBIDI, Germany) as described above. Z-sections were acquired at 0.81µm intervals on a Zeiss LSM 880 point scanning confocal microscope using the Airyscan detector, a 20x plan-apochromat 0.8 NA objective (Zeiss) and the 488nm and 561nm laser lines. The Zeiss Zen 3.0 (black edition) software was used to control the microscope, adjust spectral detection for the emission of GFP and RFP and to process the Airyscan raw images.

### Colorimetric Quantification of Biofilm Biomass Through Crystal Violet Staining

Single- and dual-strain biofilms were grown for 72h as described. At hour 72, supernatants were removed and biofilms were imaged before and after crystal violet staining. For staining, 50µL of crystal violet (CV, 1% w/v) were added to each well and incubated at room temperature for 30 min. CV was removed by inversion, the plates were allowed to air-dry for 10 min and the biofilms were washed twice with 500µL of PBS. Biofilms were imaged on a Zeiss Axio Zoom.V16 stereo microscope equipped with a Zeiss Axiocam 503 mono CCD camera and controlled with the Zen 2.1 software (blue edition), using the 1X 0.25NA objective and bright field optics. CV was solubilized by addition of 1mL of 95% ethanol and incubation with agitation until no stained culture was observed at the bottom of the well. Absorbance at 595nm was measured with a Tecan Infinite F200 plate reader. Absorbance was measured for three biofilms per strain or combination of strains.

### Statistical Analysis

GraphPad Prism 7.0 (GraphPad Software Inc., La Jolla, California, USA) was used for all statistical analyses. The Kruskall-Wallis test corrected for FDR with the Benjamini and Hochberg method was used for comparisons between three groups. The Mann-Whitney U test was used for comparisons between two groups. The Mann-Whitney U test corrected for FDR with the Benjamini and Hochberg method was used for the remaining comparisons.

## Results

### Fluorescence Labelling of Epidemiologically Relevant *S. pneumoniae* Strains

**Figure 1:**
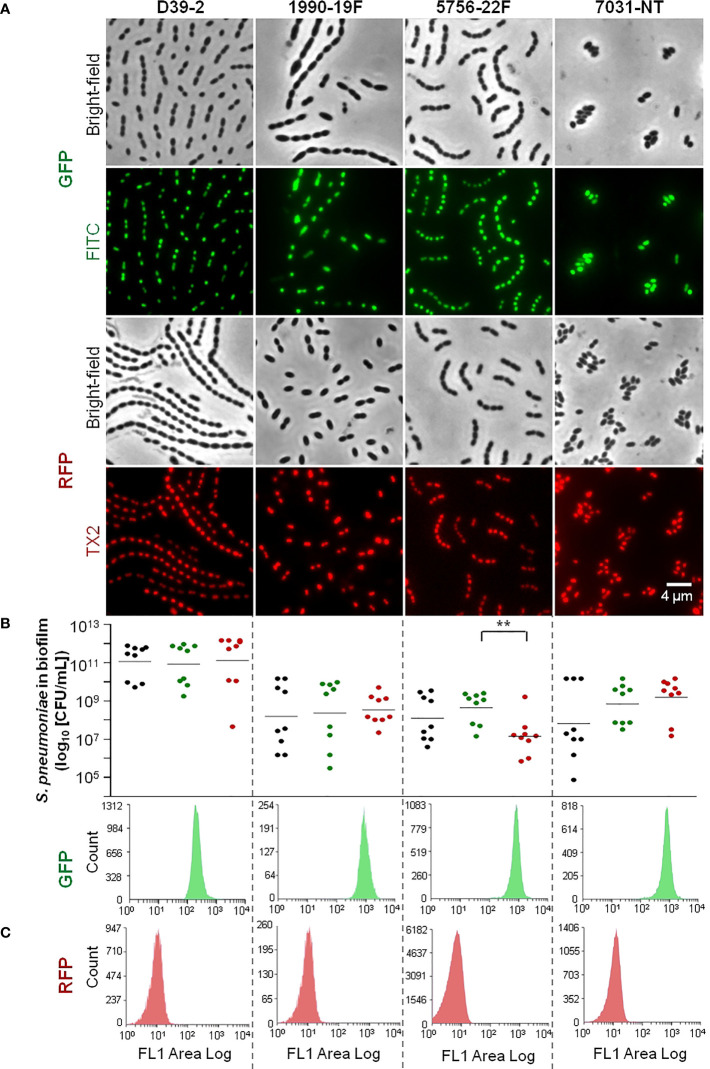
Representative fluorescently labelled strains used in the study (remaining strains are shown in **Figure S1**). Strains’ reference and serotype are indicated on top of panel **(A)**. **(A)** Imaging of GFP and RFP-labelled strains by fluorescence microscopy. GFP- and RFP-labelled strains were grown until exponential phase and imaged by fluorescence microscopy with filters for contrast phase optics, FITC and TX2. **(B)** Viability of wild-type and fluorescently labelled variants in the biofilm model. Each WT strain and its correspondent GFP- and RFP-labelled variants were grown under biofilm-promoting conditions (see the *Materials and Methods* section), after which viability (CFU/mL) was determined. Black, green and red dots indicate WT, GFP- and RFP-labelled cells, respectively. three independent experiments, each with an intra-experiment triplicate. **p < 0.01 (Kruskal Wallis test with Benjamini and Hochberg correction for FDR). **(C)** Flow cytometry analysis of fluorescent variants in biofilm model. Biofilm formation was induced as described in **(B)**. After 72h biofilms were resuspended and sonicated and cell counts were obtained by flow cytometry. Representative histograms are shown.

We were able to successfully obtain GFP- and RFP-labelled variants of 14 pneumococcal strains of nine serotypes (serotypes 2, 3, 4, 15A, 19A, 19F, 22F, 33F, and NT) and 12 genetic backgrounds (STs 63, 177, 205, 260, 276, 344, 433, 595, 717, 4583, 8322, and 9161) (**Table S2**). Fluorescence microscopy allowed confirmation of GFP or RFP expression in all cells of the population for each fluorescent variant (**Figures 1A**, **S1**). The exceptions to this scenario were the RFP-labelled variant of strain 5262.1-19A and the GFP-labelled variant of strain 2099-3 in which fluorescence was noted in a small fraction of the population only (**Figure S1**, see, for example, cells marked with arrow).

We were unable, however, to transform eight other strains initially selected to be included in the study. These strains had the serotype-ST combinations 1-228, 11A-408, 15A-63, 19F-309, 33F-717, 35B-198, NT-3097, and NT-6996. Transformation of these strains was attempted multiple times using planktonic and biofilm conditions. Possibly, the laboratory conditions may have been inadequate to induce competence in these strains or the strains might have defects in genes essential for competence (Lee and Morrison, 1999; Croucher et al., 2011). Other studies have also reported difficulties in transforming non-laboratory strains (Joloba et al., 2010; Evans and Rozen, 2013).

### Cell Viability and Population Homogeneity of *S. pneumoniae* GFP- and RFP-Labelled Variants

In preparation for dual-strain biofilm experiments mixing GFP- and RFP-labelled strains, we investigated, for each labelled variant, the cell viability and the homogeneity of the fluorescence signal in 72h biofilms.

Single strain biofilms were prepared and cell viability (CFU/mL) of the WT (unlabelled) strain and the corresponding GFP- or RFP-labelled variants was compared. Strains displayed different abilities to grow in biofilm, with mean CFU counts at 72h ranging between 10^5^ and 10^11^ CFU/mL (p<0.001, Kruskal-Wallis test with Benjamini and Hochberg correction for False Discovery Rate). For most triplets (9 of 14) no significant differences were observed (**Figures 1B**, **S1**). One triplet (corresponding to WT strain 5756) showed significant differences in the viability of labelled variants (GFP vs RFP), but no significant differences between each variant and the WT (**Figure 1B**). The other four triplets (corresponding to WT strains 2099, 5262.2, 5902, and 8046) showed significant differences in cell viability (in at least one variant when compared to the WT strain) and were excluded from further analyses (**Figure S1**).

We then wanted to identify GFP and RFP variants yielding homogenous (narrow single peaks) and non-overlapping profiles, an essential requirement to enable cell counts of two populations of the same species by flow cytometry. With that in mind, the distribution of the fluorescence signal in the population was investigated by flow cytometry, for each strain variant (GFP or RFP), after growth of single-strain biofilms. For most variants, biofilm cells produced a homogenous signal (**Figures 1C**, **S1**). For two strains (5262.1 and 8276), however, high heterogeneity was observed for both the GFP and RFP signals (**Figure S1**). As this would compromise the capacity to distinguish mixed GFP- and RFP-producing cultures, these variants were excluded.

In summary, we selected GFP- and RFP- variants of eight strains to be used in dual-strain biofilm experiments.

### Dual-Strain Biofilm Experiments

**Figure 2:**
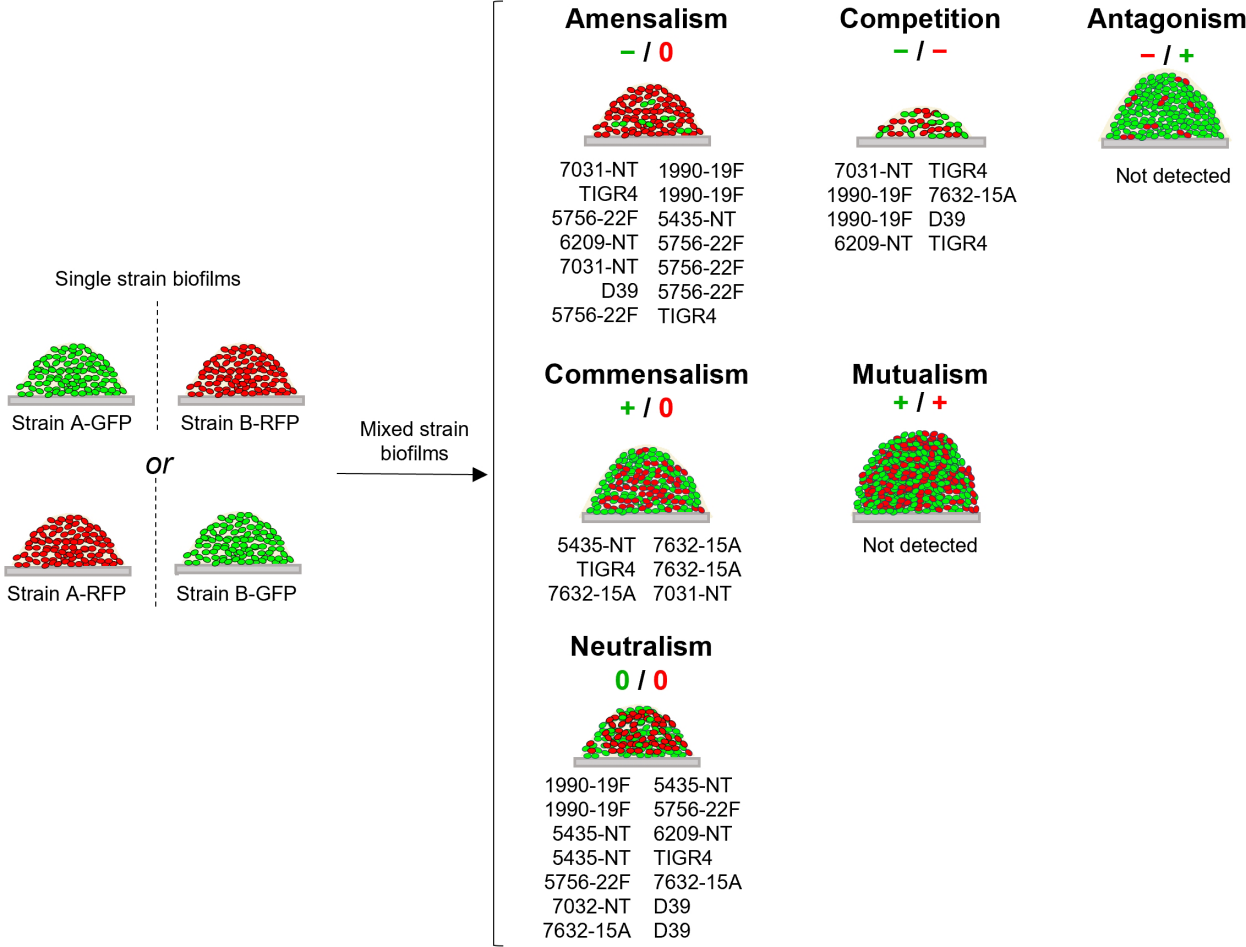
Schematic representation of interactions identified among pneumococcal strains. For each strain growing in a dual-strain biofilm three possible effects can be observed when its cell counts are compared to those obtained when growing alone (single-strain biofilms depicted on the left): negative (-), if there is a decrease in its cell counts; positive (+), if there is an increase in its cell counts; and neutral (0), if there is no change. Depending on the combination of effects observed for each pair of strains, interactions can be divided into six categories: neutralism (0/0), mutualism (+/+), commensalism (+/0), antagonism (-/+), amensalism (-/0) and competition (-/-) [reviewed in (Faust and Raes, 2012)]. The interaction observed for each pair of strains is indicated below the schematic representation of each interaction.

The eight strains remaining in the study were grown in dual-strain biofilms in which one strain was labelled with GFP and the other with RFP (for example, strain A-GFP was grown together with strain B-RFP), for a total of 28 combinations. As controls, the reverse labelling combination was also tested in a dual-strain biofilm (A-RFP together with B-GFP), and single-strain biofilms of all variants were also examined (A-GFP, A-RFP, B-GFP, and B-RFP) (**Figure 2**). In all cases, cell counts for each strain were measured at 72h by flow cytometry.

For most combinations (21 out of 28), no significant differences in cell counts were observed when the isogenic GFP-and RFP-variants of a given strain were compared in the same experimental condition (**Figures S2A–D** ). In seven combinations significant variations were observed in at least one comparison, in which case the assays were excluded from further analyses (**Figure S2E**).

In total, twenty-one combinations of dual-strain biofilms were validated for the investigation of intra-species interactions. These included strains of serotypes 2, 4, 15A, 19F, 22F, and NT of six distinct MLST profiles (**Table 2** and **Figure S2**).

### Identification of Intra-Species Interactions

To identify intra-species interactions, cell counts of each strain grown in dual-strain biofilm were compared to those obtained in a single-strain biofilm experiment run in parallel. Among the 21 combinations of dual-strain biofilms tested, interactions of commensalism (n=3), competition (n=4), amensalism (n=7) and neutralism (n=7) were detected (**Table 3** and **Figure 2**, **S2**).

**Table 3:** Dual-strain combinations tested for intra-species interactions.

Strains tested	Cell counts in dual biofilm^a^ (GM dual)	Cell counts in single biofilm^a^ (GM single)	GM dual^b^	Net result^c^	Type of interaction
			GM single		
**5435-NT and 7632-15A**					
5435-NT	5.37x10^7^	4.28x10^5^	1.25x10^2^	↑	Commensalism
7632-15A	2.05x10^8^	8.10x10^8^	Ns	=	
**7632-15A and TIGR4**					
7632-15A	4.44x10^6^	7.26x10^6^	Ns	=	“
TIGR4	9.54x10^5^	2.06x10^5^	4.64x10^0^	↑	
**7031-NT and 7632-15A**					
7031-NT	1.13x10^7^	1.86x10^5^	ns	=	“
7632-15A	2.97x10^8^	1.07x10^8^	2.77x10^0^	↑	
**7031-NT and TIGR4**					
7031-NT	3.08x10^4^	1.03x10^6^	3.00x10^-2^	↓	Competition
TIGR4	5.51x10^4^	2.73x10^5^	2.02x10^-1^	↓	
**1990-19F and 7632-15A**					
1990-19F	6.87x10^4^	1.12x10^6^	6.12x10^-2^	↓	“
7632-15A	2.84x10^6^	7.00x10^6^	4.06x10^-1^	↓	
**1990-19F and D39**					
1990-19F	2.92x10^5^	1.21x10^6^	2.41x10^-1^	↓	“
D39	1.83x10^4^	2.53x10^5^	7.22x10^-2^	↓	
**6209-NT and TIGR4**					
6209-NT	1.66x10^5^	3.46x10^6^	4.80x10^-2^	↓	“
TIGR4	5.07x10^5^	4.49x10^6^	1.13x10^-1^	↓	
**1990-19F and 7031-NT**					
1990-19F	2.23x10^6^	2.29x10^6^	ns	=	Amensalism
7031-NT	1.18x10^4^	2.66x10^7^	4.44x10^-4^	↓	
**1990-19F and TIGR4**					
1990-19F	1.24x10^5^	2.98x10^5^	ns	=	“
TIGR4	1.99x10^4^	2.10x10^5^	9.47x10^-2^	↓	
**5435-NT and 5756-22F**					
5435-NT	7.05x10^6^	8.23x10^6^	ns	=	“
5756-22F	4.68x10^7^	8.94x10^7^	5.23x10^-1^	↓	
**5756-22F and 6209-NT**					
5756-22F	1.46x10^7^	2.03x10^7^	ns	=	“
6209-NT	1.39x10^6^	4.78x10^6^	2.92x10^-1^	↓	
**5756-22F and 7031-NT**					
5756-22F	1.84x10^7^	1.25x10^7^	ns	=	“
7031-NT	1.56x10^6^	1.30x10^7^	1.20x10^-1^	↓	
**5756-22F and D39**					
5756-22F	5.46x10^5^	9.62x10^5^	ns	=	“
D39	4.61x10^5^	1.07x10^6^	4.31x10^-1^	↓	
**5756-22F and TIGR4**					
5756-22F	3.60x10^4^	1.44x10^6^	2.49x10^-2^	↓	“
TIGR4	1.57x10^5^	2.28x10^5^	ns	=	
**1990-19F and 5435-NT**					
1990-19F	2.49x10^5^	5.77x10^5^	ns	=	Neutralism
5435-NT	8.41x10^4^	9.92x10^4^	ns	=	
**1990-19F and 5756-22F**					
1990-19F	7.80x10^5^	1.77x10^6^	ns	=	“
5756-22F	4.33x10^5^	5.84x10^5^	ns	=	
**5435-NT and 6209-NT**					
5435-NT	1.13x10^5^	4.83x10^5^	ns	=	“
6209-NT	6.88x10^6^	7.96x10^6^	ns	=	
**5435-NT and TIGR4**					
5435-NT	7.62x10^4^	1.27x10^5^	ns	=	“
TIGR4	7.40x10^4^	6.79x10^5^	ns	=	
**5756-22F and 7632-15A**					
5756-22F	4.84x10^6^	3.55x10^6^	ns	=	“
7632-15A	3.89x10^7^	1.74x10^7^	ns	=	
**7031-NT and D39**					
7031-NT	3.57x10^5^	2.79x10^6^	ns	=	“
D39	7.61x10^5^	1.62x10^6^	ns	=	
**7632-15A and D39**					
7632-15A	2.25x10^6^	6.16x10^6^	ns	=	“
D39	7.58x10^5^	3.05x10^5^	ns	=	

^a^Cell counts, as a proxy of cell viability, were obtained by flow cytometry. Values indicate the geometric mean of 18 biological replicates (nine with GFP-labelling and nine with RFP-labelling) obtained for each strain in each condition (single-strain or dual-strain biofilm). The detailed results obtained for biological replica are presented in **Figure 3** and **Figure S2**.

b The ratio between the geometric mean of cell counts of each strain in dual- and single-strain biofilms was calculated as a proxy of the strength of intra-species interaction. Results are only indicated when statistically significant differences were obtained following comparisons of the cell counts in dual- vs single-strain biofilms.

c ↑ strain benefits (i.e., cell counts increase) from growing in the presence of the other strain; ↓ strain is harmed (i.e., cell counts decrease) when growing in the presence of the other strain; = strain is not affected by the presence of the other strain.

ns, not significant.

Importantly, we found that the outcome of an interaction is independent of the capsular and sequence types of the strains involved. An example that illustrates this observation is the interaction of strain 7632-15A (ST8322) with strains 5435-NT and 7031-NT (both ST344). In the first case (7632-15A and 5435-NT) the NT strain is benefited by the presence of the 15A strain, whereas in the second case (7632-15A and 7031-NT) it is the 15A strain that is benefited by the presence of the NT strain.

### Imaging of Intra-Species Interactions Occurring in Dual-Strain Biofilms

**Figure 3:**
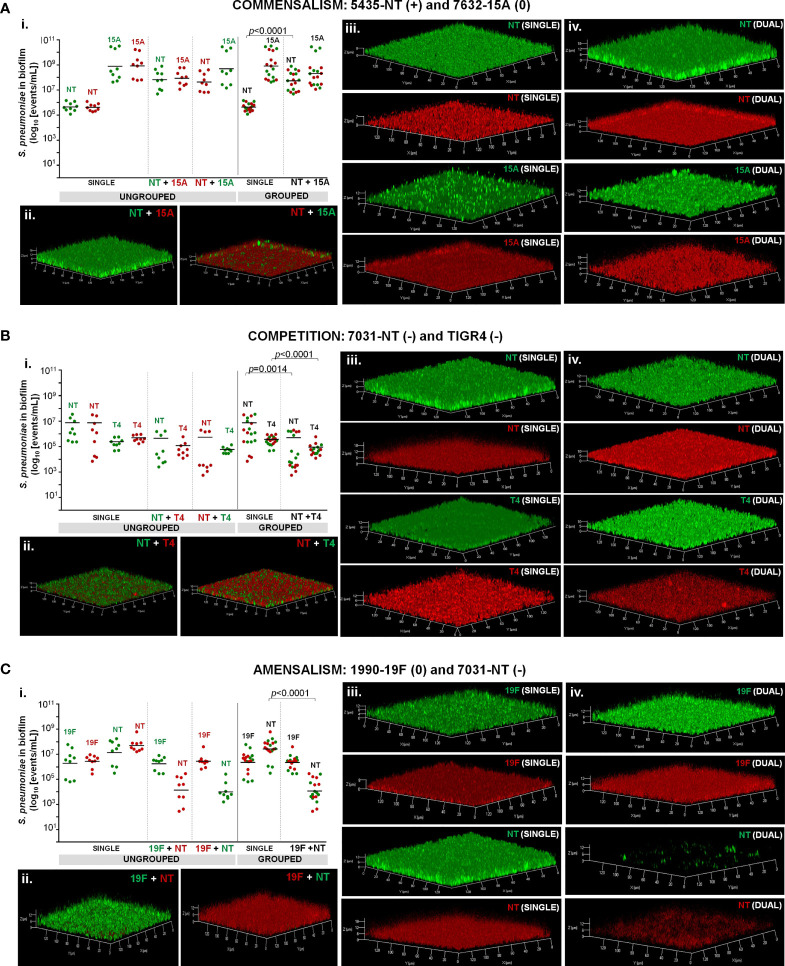
Dual-strain combinations representative of commensalism **(A)**, competition **(B)**, and amensalism **(C)**. Strains were tested in dual-strain biofilms (one GFP- and one RFP-labelled strain) in a 1:1 ratio. Reverse labelling combinations were also tested as controls. For comparative analysis, single-strain biofilms were grown in parallel. Intra-species interactions were considered to occur when statistically significant differences between a given strain in single and dual-strain biofilms were observed (p<0.05), Mann-Whitney U test corrected with Benjamini and Hochberg method for FDR. (i.) Cell counts. Cell counts of strains in single- and dual-strain biofilms were obtained by flow cytometry, events/mL were calculated and compared. For each strain, one out of three possible outcomes was determined when grown in the presence of another: positive (+), if there was beneficial effect; neutral (0), if there was no effect; and negative (-) if there was harm. Black bars indicate geometric means. Green and red dots correspond to GFP- and RFP-labelled cells, respectively. Graphics represent the ungrouped and grouped analyses for each pair using data from three independent experiments, each with an intra-experiment triplicate. (ii.) Imaging of dual-strain biofilms. (iii.) Imaging of single strain biofilms (iv.). Single-channel images of dual-strain biofilms. All CLSM images are transparent projections of 3D reconstructions from z-sections acquired at 0.81 µm intervals on a Zeiss LSM 880 point scanning confocal microscope using the Airyscan detector, a 20x plan-apochromat 0.8 NA objective (Zeiss) and the 488 nm and 561 nm laser lines.

Dual-strain biofilms representative of interactions of commensalism, competition, and amensalism were selected for confocal microscopy. Imaging of single- and dual-strain biofilms produced by strains 5435-NT and 7632-15A confirmed the commensality identified between these two strains by flow cytometry (**Figure 3A**). In particular, we confirmed that strain 5435-NT increased in biofilm depth (*z-axis*, proxy to biofilm biomass) when in co-culture with strain 7632-15A (**Figure 3A** and **Table S3**).

Similarly, the competitive interaction observed by flow cytometry for strains 7031-NT and TIGR4 was also detected in the confocal microscopy images (**Figure 3B**). A decrease in biofilm depth was noted for both strains when grown in dual-strain biofilms as compared to their growth in single-strain biofilm (**Figure 3B** and **Table S3**).

Finally, the amensalism identified between strains 1990-19F and 7031-NT by flow cytometry (**Figure 3C**) was also observed by confocal microscopy imaging of these two strains when grown in single- and dual-strain biofilms. The significant decrease in cell counts (quantified by flow cytometry) of strain 7031-NT when growing in the presence of strain 1990-19F, translated into a major reduction of the biofilm produced by this strain when in co-culture with the 19F strain. In fact, the total biofilm biomass of the dual-strain biofilms of this pair was found to be mostly attributable to the growth of the 19F strain (**Figure 3C** and **Table S3**).

The same representative pairs were also imaged before and after crystal violet staining (**Figure S3A**) and the total biofilm biomass was quantified (**Figure S3B**). The biomass quantified following crystal violet staining correlated with the biomass results obtained using CLSM.

### Imaging of Intra-Species Interactions Occurring in Dual-Strain Biofilms Grown in Different Concentrations of Choline

To rule out possible effects of the choline concentration used in our mCDM (1mg/mL) on the activity of choline-binding proteins – important for pneumococcal growth in biofilm and for fratricide (Moscoso et al., 2006; Perez-Dorado et al., 2010) -, single- and dual-strain biofilms representative of the interactions identified were grown in low (4µg/mL) and high (20mg/mL) concentrations of choline chloride.

At a lower concentration of choline (4µg/mL) there was lower biofilm growth, both in single- and dual-strain biofilms. Still, the interactions previously observed were maintained (**Figure S4**). By contrast, the increase of choline chloride to 20mg/mL severely hampered the ability of strains to grow in biofilm (in agreement with previous studies focusing on the importance of CBPs to biofilm development) (Moscoso et al., 2006). This lack of growth in biofilm prevented us to draw conclusions on the effect of this high concentration of choline in the interactions detected (**Figure S5**). Taken together, the results showed that the concentration of choline chloride used in our mCDM promotes better biofilm growth and has no impact on the interactions observed.

## Discussion

Multiple strains of pneumococci often coexist in the upper respiratory tract (Turner et al., 2011; Valente et al., 2012; Kamng’ona et al., 2015; Kandasamy et al., 2015; Valente et al., 2016b). The intra-species interactions resulting from this co-existence, however, have not been actively investigated. We developed a strategy to study such interactions and applied it to a diverse collection of *S. pneumoniae* strains.

We tested eight pneumococcal strains in pairs in a three-day long abiotic biofilm model. We found that, depending on the combination, strains could be positively affected, negatively affected or unaffected by the presence of other strain. Four types of interactions were observed: commensalism (14%), competition (19%), amensalism (33%), and neutralism (33%).

All positive interactions were of commensalism. To the best of our knowledge, this is the first report describing commensalism between naturally-occurring, colonizing strains of *S. pneumoniae*. We observed that strains of diverse backgrounds were positively affected, i.e. produced a better biofilm, in the presence of other strain.

Positive interactions between strains of the same species were identified before (Selak et al., 2016). Selak et al. showed that natural isolates of bifidobacteria obtained from intestinal biopsies cooperated for the degradation of complex carbohydrates. This resulted in cross-feeding and co-existence of strains with different metabolic profiles enabling colonization of diverse areas of the intestinal lumen (Selak et al., 2016). Since nutrient diffusion within biofilms is diminished (Stewart, 2003), it seems possible that the positive interactions identified in our study result, at least in part, from a mechanism of cross-feeding of metabolic by-products or nutrients resulting from cell lysis. Another possibility is that cooperation is occurring through the production of molecules that enable scavenging of otherwise unavailable nutrients or that degrade specific metabolites. We are conducting additional studies to address these possibilities.

Negative interactions were due to competition or amensalism. Both types of interactions were observed before in pneumococci. Pneumococcal colonization studies in children have repeatedly reported that, at any time, only a few serotypes account for most of the isolates, suggesting strain competition for niche occupancy affecting population structure and epidemiology (Félix et al., 2021). Biofilm studies and experiments with animal models have also unravelled these types of interactions and have explored some of the biological mechanisms governing them (Lipsitch et al., 2000; Dawid et al., 2007; Trzciński et al., 2015; Shen et al., 2019). Examples of such mechanisms include bacteriocin secretion (Dawid et al., 2007; Maricic et al., 2016) and exclusion of a newcomer by a resident strain *via* competence-mediated fratricide (Shen et al., 2019). Mechanisms of passive competition, where one strain is indirectly affected by the presence of another, could also have a role in the negative interactions identified in our study. Differences in colonization ability attributable to the capsular type metabolic cost and associated cell surface charge (Lipsitch et al., 2000; Trzciński et al., 2015), in growth rates, or in tolerance to metabolic products or niche components, for example, could be involved.

Neutral interactions were detected in one third of the combinations tested indicating that either pneumococcal strains frequently co-exist without affecting each other (neutralism), or that those effects could not be detected with the system used. Neutral interactions among bacteria are not frequently described. Nevertheless, a recent study reported dominance of neutral interactions among actinobacteria in the soil ecosystem (Yan et al., 2021), particularly in oligotrophic conditions*, i.e.*, in nutrient poor conditions that resulted from metabolic overlap between species. Given the large array of metabolic profiles identified among pneumococcal strains (Watkins et al., 2015), it seems possible that our strains have different nutritional requirements; this possibility, however, was not addressed in the present study. It is also possible that intra-species interactions may occur at a local level, with no impact on the total cells that form the biofilm (Kim et al., 2014). As populations of cells are being studied as a whole, such interactions would also have been missed with our approach.

Our study has some limitations. Firstly, our experimental approach was based on an *in vitro* system that does not take into consideration the effect that host- or microbiota-associated factors might have on pneumococcal intra-species interactions. In addition, our strains were inoculated in equal proportions and at the same time point. These are important limitations as, in natural colonization, pneumococcal strains co-occurring in the same host often are present at different proportions and may have been acquired in different occasions (Valente et al., 2012; Valente et al., 2016b). This approach prevented us from detecting positive or negative interactions that are dependent on relative cell-density and time of strains’ exposure. An example where this scenario would be relevant is the competition model proposed by Shen and co-authors, in which earlier activation of the competence regulon, and hence the production of the fratricide effectors CbpD and CibAB, conferred an advantage to the resident strain (occupying a niche) in the presence of a newcomer (Shen et al., 2019).

Secondly, some GFP- and RFP- labelled variants showed significant differences in cell viability compared to the parental strain; others had significant heterogeneity in the fluorescence signal. These limitations prevented the use of some strains with our experimental approach. Both fluorescent constructs used in this study were expressed under the control of the *hlpA* promoter. Kjos et al. showed that for the laboratory strain D39, *hlpA-gfp* and *hlpA-rfp* fusions did not affect cell viability nor growth rate in liquid medium, were expressed throughout the cell cycle, and that fluorescence intensity remained high and stable with low cell-to-cell variation, as assessed by fluorescence microscopy (Kjos et al., 2015). Variation in the fluorescence signal, however, was not tested under biofilm-forming conditions, nor inspected for other strains or evaluated by flow cytometry. Possibly, the heterogeneity observed in our strains derives from natural variability previously described for isogenic strains (reviewed in (Jørgensen et al., 2013)), from the existence of a sub-population of cells that are either dying or dead but still retaining cell integrity (biofilms include many dead cells (Bayles, 2007)), or a combination of both. These limitations led to the exclusion of various strains limiting the magnitude of potential findings.

Thirdly, we observed heterogeneity within replicated biofilm experiments, which even though expected, hampered analyses of some dual-strain specific combinations. Marks et al. have shown that biofilms grown on an epithelial cell layer have increased reproducibility and lead to higher biofilm yields compared to biofilms obtained in abiotic conditions (Marks et al., 2012). In our optimized abiotic system, we have successfully obtained yields comparable to the ones described by Marks et al. The inclusion of an epithelial cell layer, albeit possibly increasing reproducibility, would increase the complexity of the analysis and would hinder accurate distinction and quantification of pneumococcal strains by flow cytometry.

In any event, the use of several strains led us to make two important observations: (i) the outcome of an interaction is independent of capsular and sequence type; (ii) a given strain, when grown in the presence of another, may be benefited, harmed or not affected, depending on the strain it is interacting with.

Overall, we were able to develop a strategy that enables identification of different types of intra-species interactions in biofilms and successfully applied it to a diverse collection of pneumococcal isolates. We validated the use of flow cytometry for direct quantification of bacteria within the biofilm, as opposed to more traditional methods, such as bacterial cell counts on selective media. Our approach enables discrimination between strains of the same serotype, genotype and antibiotype, overcoming a limitation of strategies previously described (Wu et al., 2017). Representative interactions were further confirmed through a qualitative approach based on CLSM, *i.e.*, we were able to visualize positive, negative and neutral effects that *S. pneumoniae* strains may exert on each other and these were in agreement with the results obtained using flow cytometry. Furthermore, this approach could be adapted to other species and to more complex systems through the optimization of fluorescent constructs and the selection of other fluorescent reporters. In fact, a comparable strategy has recently been applied to visualize oral streptococcal species using integrative and replicative plasmids with strong promoters fused to fluorescent reporters (Shields et al., 2019).

In conclusion, we provide novel evidence that a panoply of intra-species interactions occur between naturally colonizing pneumococcal isolates and these are independent of serotype and genotype. These observations are a starting point to study the mechanisms underlying those interactions. Such studies will add to the growing body of knowledge regarding how the upper respiratory tract microbiota is shaped and adapts to changes triggered by the use of vaccines and antibiotics allowing the design of alternative strategies to modulate this ecosystem as a way to prevent infection.

## Data Availability Statement

The original contributions presented in the study are included in the article/**Supplementary Material**. Further inquiries can be directed to the corresponding author.

## Author Contributions

The study was designed by CV and RS-L. Data acquisition, analysis and interpretation were performed by CV, AC, AH, and RS-L. The manuscript was drafted by CV, AC, and RS-L and critically revised by all authors. All authors contributed to the article and approved the submitted version.

## Funding

This work was supported by project PTDC/BIA-MIC/30703/2017 from Fundação para a Ciência e a Tecnologia (FCT), Portugal; LISBOA-01-0145-FEDER-007660 (Microbiologia Molecular, Estrutural e Celular, funded by FEDER through COMPETE2020 – Programa Operacional Competitividade e Internacionalização); and LISBOA-01-0145-FEDER-016417 (ONEIDA co-funded by Fundos Europeus Estruturais e de Investimento, Programa Operacional Regional Lisboa 2020 and Fundação para a Ciência e a Tecnologia (FCT)). CV was supported by post-doctoral fellowship from FCT SFRH/BPD/115280/2016. The funders had no role in the design of the study, collection, analysis, and interpretation of data, writing of the manuscript or in the decision to submit the manuscript for publication. The work was partially supported by PPBI - Portuguese Platform of BioImaging (PPBI-POCI-01-0145-FEDER-022122) co-funded by national funds from OE - “Orçamento de Estado” and by European funds from FEDER - “Fundo Europeu de Desenvolvimento Regional”.

## Conflict of Interest

The authors declare that the research was conducted in the absence of any commercial or financial relationships that could be construed as a potential conflict of interest.

## Publisher’s Note

All claims expressed in this article are solely those of the authors and do not necessarily represent those of their affiliated organizations, or those of the publisher, the editors and the reviewers. Any product that may be evaluated in this article, or claim that may be made by its manufacturer, is not guaranteed or endorsed by the publisher.
