# Supplementary material for: Intra-Species Interactions in Streptococcus pneumoniae Biofilms
Source: Front Cell Infect Microbiol. 2022 Jan 5;11:803286. doi: 10.3389/fcimb.2021.803286 (PMC8767070; doi:10.3389/fcimb.2021.803286)
Supplement: Supplementary file 1 [file DataSheet_1.pdf]

SUPPLEMENTARY MATERIAL - TABLES

Table S1. Composition of modified chemically defined medium (mCDM)

Component	Concentration (mg L^{-1})
**Phosphate buffer**	
K_2HPO_4	200
KH_2PO_4	1000
$NaH_2PO_4 \cdot H_2O$	3200
$Na_2HPO_4 \cdot 2H_2O$	6936
**Metals**	
$MnSO_4$	5
$MgSO_4 \cdot 7H_2O$	700
$CaCl_2$	5.1
$Fe(NO_3)_3 \cdot 9H_2O$	1
Fe $SO_4 \cdot 7H_2O$	5
**Bases**	
Adenine	20
Guanine hydrochloride	20
Uracil	20
**Vitamins**	
p-Amonibenzoic acid	0.2
Biotin	0.2
Folic Acid	0.8
Niacinamide	1
β-Nicotinamide adenine nucleotide	2.5
Pantothenate calcium salt	2
Pyridoxal	1
Pyridoxamine dihydrochloride	1
Riboflavin	2
Thiamine hydrochloride	1
Vitamin B_{12}	0.1
**Amino acids**	
DL-Alanine, L-Arginine, L-Aspartic acid, L-Asparagine, L-Glutamic acid, Glycine, L-Histidine, L-Isoleucine, L-Leucine, L-Lysine, L-Methionine, L-Phenylalanine, L-Proline, Hydroxy-L-proline, L-Serine, L-Tryptophan, L-Tyrosine, L-Valine	100 of each
L-Cystine	50
L-Glutamine, L-Threonine	200 of each
**$NaHCO_3$**	2500
**$NaC_2H_3O_2$**	2714
**Cysteine HCl**[a]	800
**Choline chloride**[a]	1000
**Galactose**[a]	10000

mCDM is a modified version of the CDM proposed by Van de Rijn and Kessler (van de Rijn and Kessler, 1980). The differences are: cysteine-HCl is at higher concentration in the current version (800mg mL^{-1} instead of 500mg mL^{-1}), choline chloride was absent in the original recipe, and glucose was replaced by galactose at the same concentration.

Table S2. Fluorescent variants of the bacterial strains selected for the study of intra-species interactions.

Strain	Relevant characteristics[c]	Reference
D39_GFP	D39 *hlpA-gfp*_Cam^r	This study
D39_RFP	D39 *hlpA_hlpA-rfp*_Cam^r	This study
TIGR4_GFP	TIGR4 *hlpA-gfp*_Cam^r	This study
TIGR4_RFP	TIGR4 *hlpA_hlpA-rfp*_Cam^r	This study
2099-3_GFP[a]	2099-3 *hlpA-gfp*_Cam^r	This study
2099-3_RFP[a]	2099-3 *hlpA_hlpA-rfp*_Cam^r	This study
7632-15A_GFP	7632-15A *hlpA-gfp*_Cam^r	This study
7632-15A_RFP	7632-15A *hlpA_hlpA-rfp*_Cam^r	This study
5262.1-19A_GFP[b]	5902-19A *hlpA-gfp*_Cam^r	This study
5262.1-19A_RFP[b]	5902-19A *hlpA_hlpA-rfp*_Cam^r	This study
5902-19A_GFP[a]	5902-19A *hlpA-gfp*_Cam^r	This study
5902-19A_RFP[a]	5902-19A *hlpA_hlpA-rfp*_Cam^r	This study
1990-19F_GFP	1990-19F *hlpA-gfp*_Cam^r	This study
1990-19F_RFP	1990-19F *hlpA_hlpA-rfp*_Cam^r	This study
5756-22F_GFP	5756-22F *hlpA-gfp*_Cam^r	This study
5756-22F_RFP	5756-22F *hlpA_hlpA-rfp*_Cam^r	This study
8046-22F_GFP[a]	8046-22F *hlpA-gfp*_Cam^r	This study
8046-22F_RFP[a]	8046-22F *hlpA_hlpA-rfp*_Cam^r	This study
8276-33F_GFP[b]	8276-33F *hlpA-gfp*_Cam^r	This study
8276-33F_RFP[b]	8276-33F *hlpA_hlpA-rfp*_Cam^r	This study
5262.2-NT_GFP[a]	5262-NT *hlpA-gfp*_Cam^r	This study
5262.2-NT_RFP[a]	5262-NT *hlpA_hlpA-rfp*_Cam^r	This study
5435-NT_GFP	5435-NT *hlpA-gfp*_Cam^r	This study
5435-NT_RFP	5435-NT *hlpA_hlpA-rfp*_Cam^r	This study
7031-NT_GFP	7031-NT *hlpA-gfp*_Cam^r	This study
7031-NT_RFP	7031-NT *hlpA_hlpA-rfp*_Cam^r	This study
6209-NT_GFP	6209-NT *hlpA-gfp*_Cam^r	This study
6209-NT_RFP	6209-NT *hlpA_hlpA-rfp*_Cam^r	This study

GFP: green fluorescent protein; RFP: red fluorescent protein; HlpA: histone-like protein A; NT: non-typeable (non-encapsulated).

[a]Excluded from the study due to significant differences in cell viability in biofilm between wild type and at least one of the fluorescently labelled variants.

[b]Excluded from the study due to high heterogeneity of the GFP-labelled variant

[c] In the genotype of fluorescently labelled strains the hyphen (-) and the underscore (_) indicate a translational and a transcriptional fusion, respectively. Cam^r indicates chloramphenicol resistance (Kjos et al., 2015).

Table S3. Biofilm depth of representative strains in single and dual-strain biofilms

Interaction	Strain variant tested in single biofilm	Biofilm depth in single biofilm (μm)	Stain variants tested in dual biofilm	Biofilm depth in dual biofilm (μm)
Commensalism	5435-NT-GFP	8.10	5435-NT-GFP and 7632-15A RFP	21.77
	7632-15A-RFP	16.13		
	5435-NT-RFP	7.26	5435-NT-RFP and 7632-15A GFP	16.94
	7632-15A-GFP	14.52		
Competition	7031-NT-GFP	15.32	7031-NT-GFP and TIGR4-RFP	12.15
	TIGR4-RFP	17.01		
	7031-NT-RFP	21.06	7031-NT-RFP and TIGR4-GFP	10.53
	TIGR4-GFP	12.96		
Amensalism	1990-19F-GFP	12.10	1990-19F-GFP and 7031-NT-RFP	21.77
	7031-NT-RFP	21.06		
	1990-19F-RFP	8.06	1990-19F-RFP and 7031-NT-GFP	16.94
	7031-NT-GFP	15.32		

Single and dual-strain biofims were grown in 24-well microscope slides suitable for confocal microscopy (IBIDI, Germany) for 72h, as described. For each biofilm, 10^5 cells were inoculated per well - dual-strain biofilms were inoculated in a 1:1 ratio, in a total of 10^5 cells. In each experiment two strains were tested: one labelled with GFP and one with labelled with RFP. As a control, each pair of strains was tested with reverse fluorescent labels. For comparative analysis single-strain biofilms of the same strains were done in parallel**.** Measurements were obtained from a biofilm representative of each type of interaction and correspond to total biofilm depth, both in single and dual-strain biofilms. Z-sections were acquired at 0.81 μm intervals on a Zeiss LSM 880 point scanning confocal microscope using the Airyscan detector, a 20x plan-apochromat 0.8 NA objective (Zeiss) and the 488 nm and 561 nm laser lines. The Zeiss Zen 3.0 (black edition) software was used to control the microscope, adjust spectral detection for the emission of GFP and RFP and for processing of the Airyscan raw images.

REFERENCES

Kjos, M., Aprianto, R., Fernandes, V.E., Andrew, P.W., Van Strijp, J.A., Nijland, R., and Veening, J.W. (2015). Bright fluorescent *Streptococcus pneumoniae* for live-cell imaging of host-pathogen interactions. *J Bacteriol* 197**,** 807-818.

Van De Rijn, I., and Kessler, R.E. (1980). Growth characteristics of group A streptococci in a new chemically defined medium. *Infect Immun* 27**,** 444-448.
