# Supplementary material for: Intra-Species Interactions in Streptococcus pneumoniae Biofilms
Source: Front Cell Infect Microbiol. 2022 Jan 5;11:803286. doi: 10.3389/fcimb.2021.803286 (PMC8767070; doi:10.3389/fcimb.2021.803286)
Supplement: Supplementary file 2 [file DataSheet_2.pdf]

# SUPPLEMENTARY MATERIAL - FIGURES

**Figure S1**. **Fluorescently labelled strains used in the study**

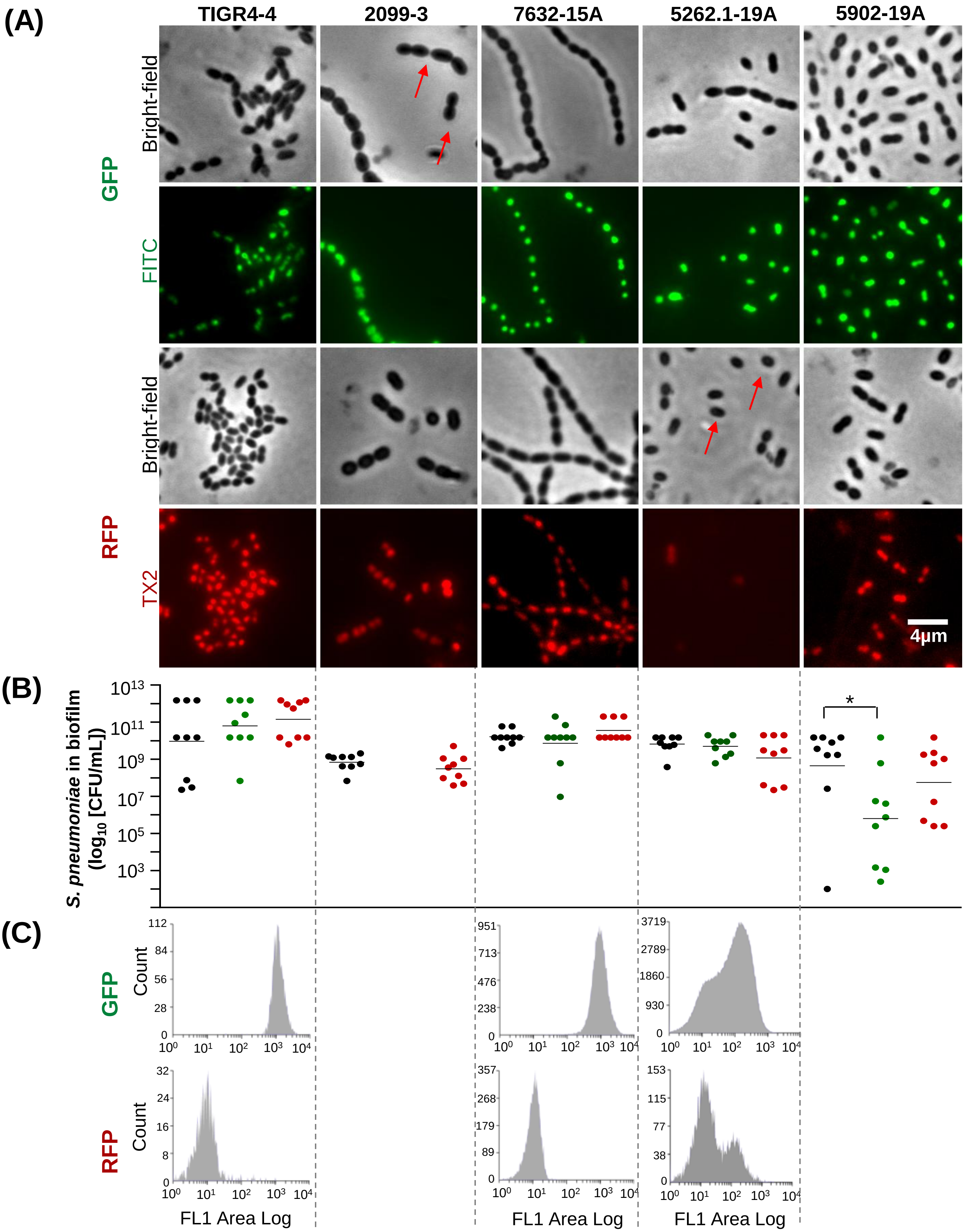

**Figure S1 (*cont.*). Fluorescently labelled strains used in the study**

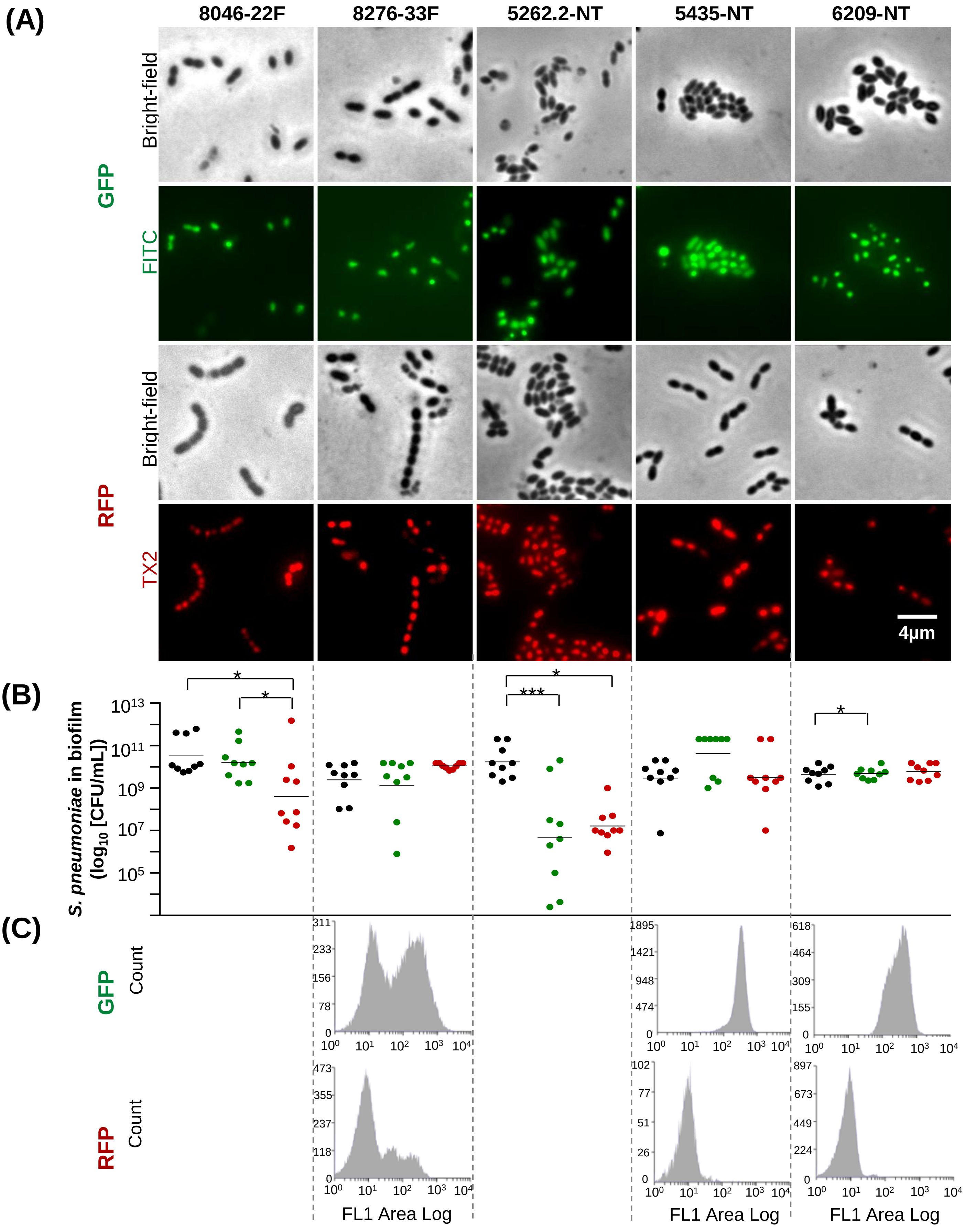

**Figure S1**. **Fluorescently labelled strains used in the study. (A) Imaging of GFP and RFP-labelled strains by fluorescence microscopy.** Strains' reference and serotype are indicated on top of panel A. GFP- and RFP-labelled strains were grown until exponential phase and imaged by fluorescence microscopy with filters for contrast phase optics, FITC and TX2. Red arrows exemplify cells that do not express fluorescence. **(B) Viability of wild-type and fluorescently labelled variants in the biofilm model.** Each WT strain and its correspondent GFP- and RFP-labelled variants were grown under biofilm-promoting conditions (see the Material and Methods section), after which viability (CFU/mL) was determined. Significant differences in viability between WT and labelled strains led to exclusion of three strains from the study (5902, 8046 and 5262.2). Strain 2099_GFP did not grow in mCDM, despite several attempts, and was also excluded. Black, green and red dots indicate WT, GFP- and RFP-labelled cells, respectively. three independent experiments, each with an intra-experiment triplicate. * $p<0.05$; ** $p<0.01$; *** $p<0.001$ (Kruskal Wallis test with Benjamini and Hochberg correction for FDR). **(C) Flow cytometry analysis of fluorescent variants in biofilm model.** Biofilm formation was induced as described in (B). After 72h biofilms were resuspended and sonicated and cell counts were obtained by flow cytometry. Representative histograms are shown. Significant heterogeneity in fluorescence expression of both fluorescent variants led to the exclusion of two strains from the study (5262.1 and 8276).

**Figure S2. Dual-strain combinations tested for intra-species interactions.**

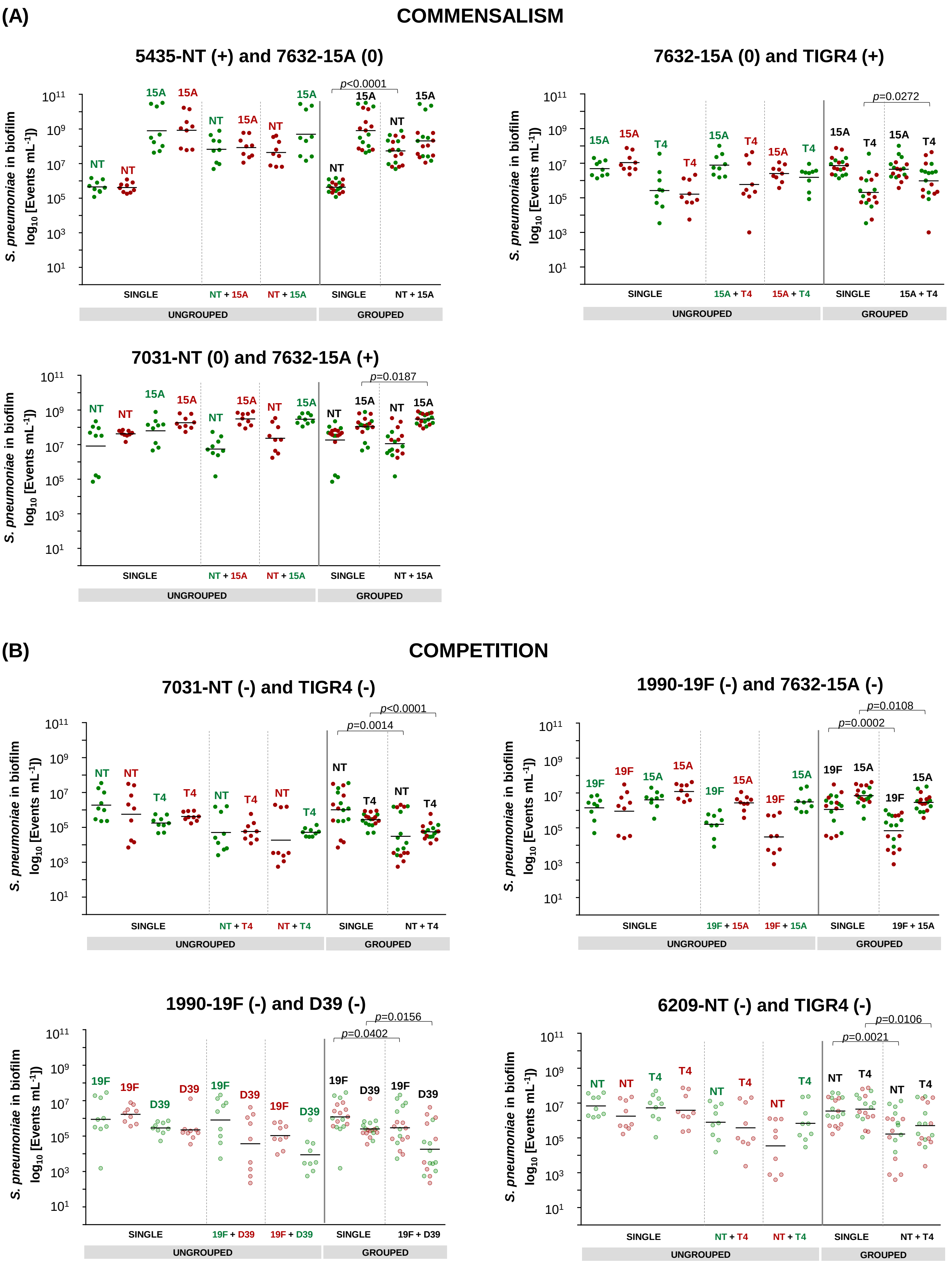

**FIGURE S2 (*cont.)* Dual-strain combinations tested for intra-species interactions.**

## (C) AMENSALISM

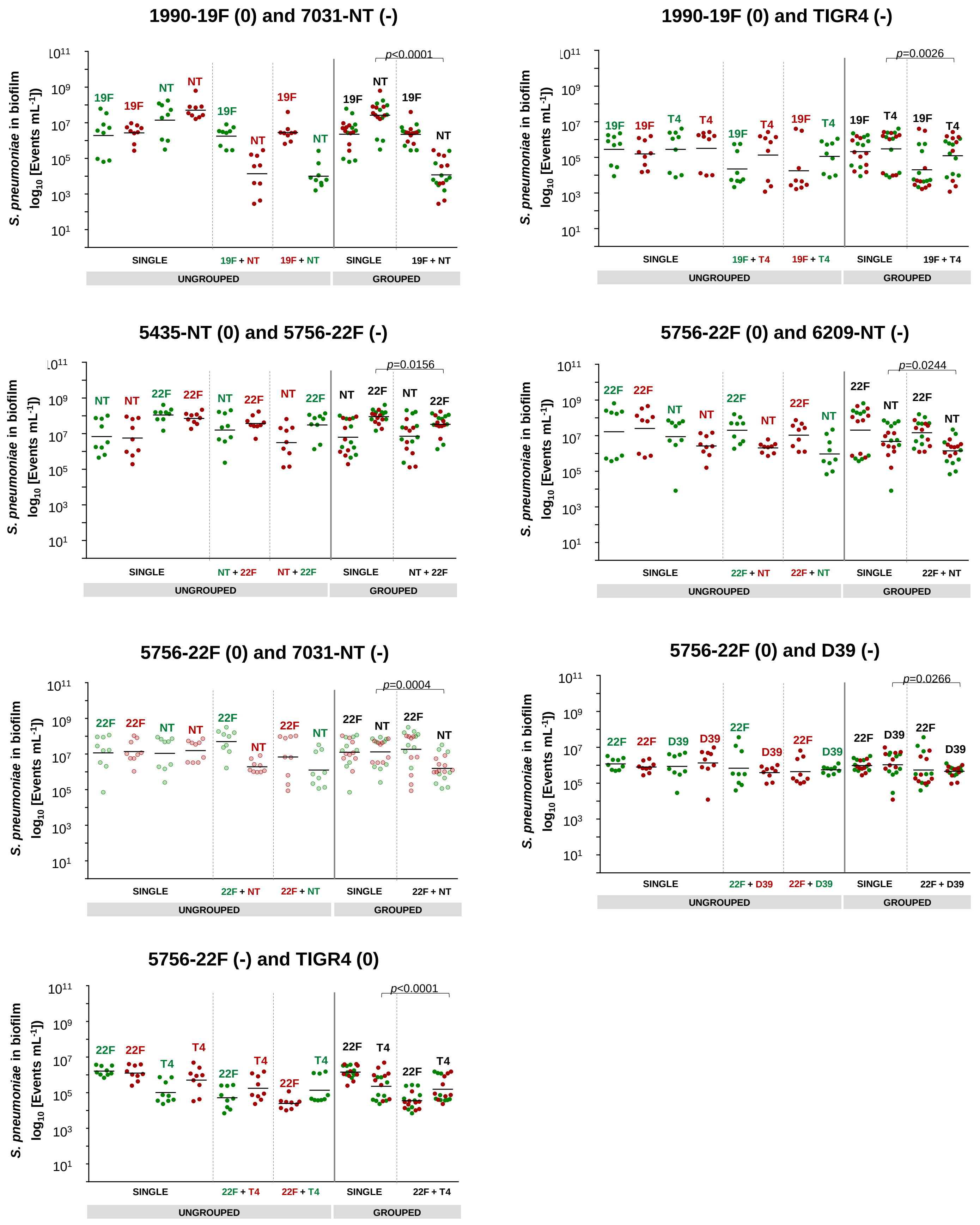

**FIGURE S2 (*cont.*). Dual-strain combinations tested for intra-species interactions.**

**(D)** **NEUTRALISM**

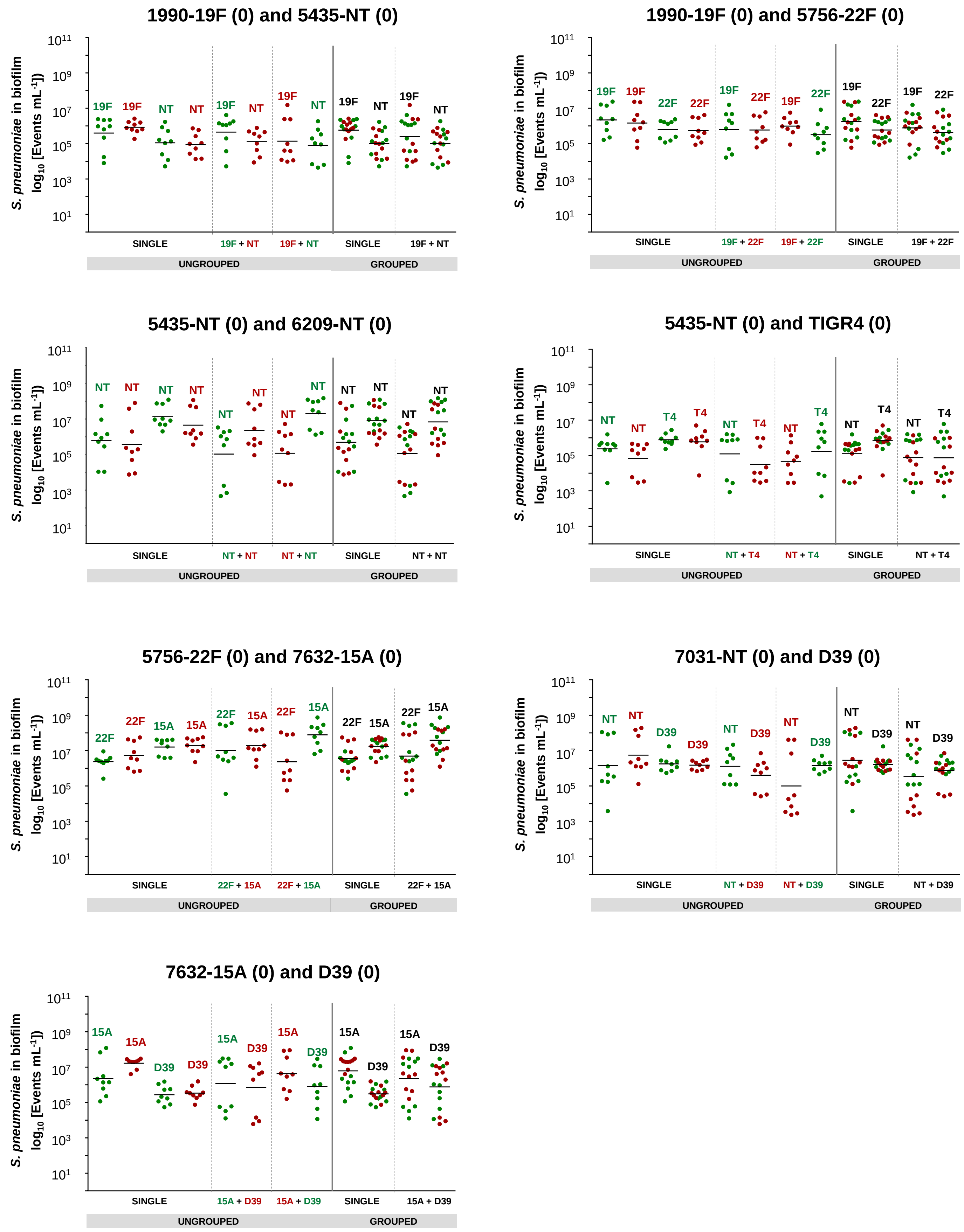

**FIGURE S2 (*cont.*). Dual-strain combinations tested for intra-species interactions.**

**(E)** 

## EXCLUDED ASSAYS

### 6209-NT and 7632-15A

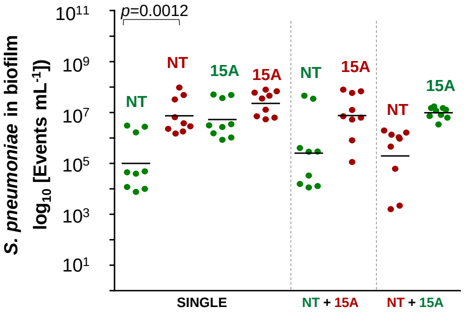


### 6209-NT and D39

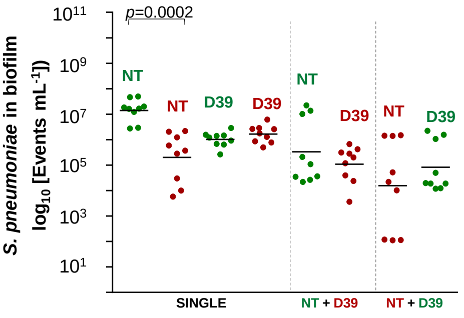


### D39 and TIGR4

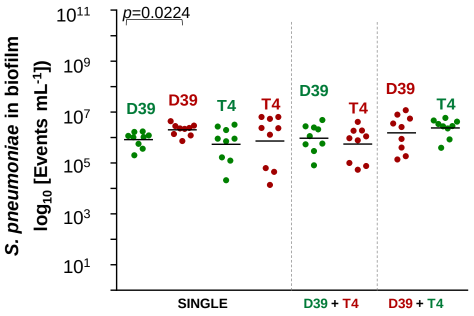


### 1990-19F and 6209-NT

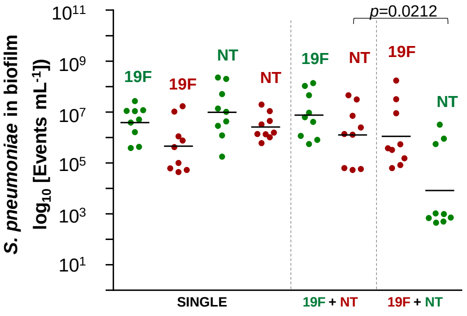


### 5435-NT and 7031-NT

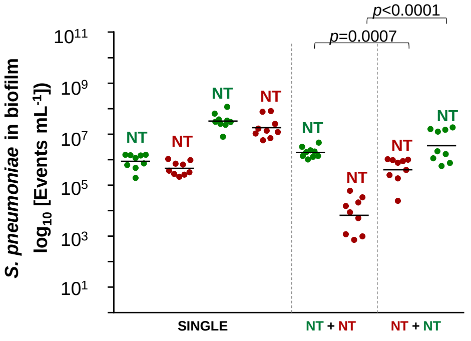


### 5435-NT and D39

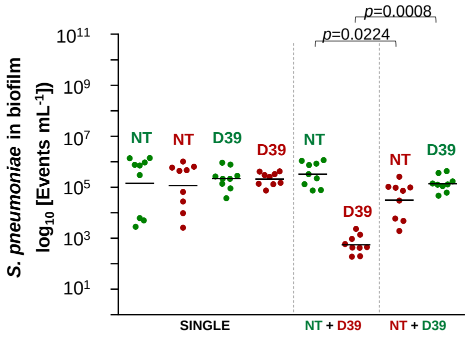


### 6209-NT and 7031-NT

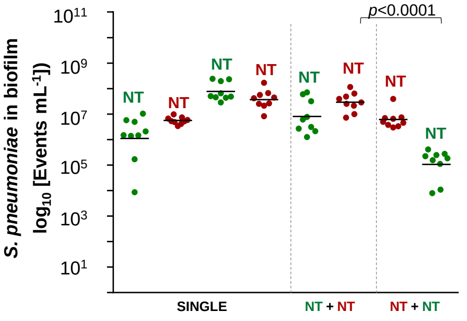

**FIGURE S3. Dual-strain combinations tested for intra-species interactions.** Strains were tested in dual strain biofilms (in a 1:1 ratio) in which one strain was labelled with GFP and the other was labelled with RFP. As a control, each pair of strains was also tested with reverse fluorescent labels. For comparative analysis, single-strain biofilms of the same strains were grown in parallel. Cell counts of strains in single- and dual-strain biofilms were obtained by flow cytometry, events/mL were calculated and compared. Intra-species interactions were considered to occur when statistically significant differences between a given strain in single and dual-strain biofilms were observed ($p<0.05$). For each strain one out of three possible outcomes was determined: positive (+), if the strain benefited from the presence of the other strain; neutral (0), if the other strain had no effect; negative (-) if the strain was negatively affected by the presence of the other strain. **Panels A-D** dual-strain combinations where interactions of commensalism, competition, amensalism, and neutralism, respectively, were observed; **panel E** dual-strain combinations where significant differences in cell counts of GFP- and RFP-labelled variants of the same strain were observed. These assays were not further analysed. Black bars indicate geometric means. Green and red dots correspond to GFP- and RFP-labelled cells, respectively. Graphics represent the ungrouped and grouped analyses for each pair using data from three independent experiments, each with an intra-experiment triplicate. Results were considered statistically significant when $p<0.05$ (Mann-Whitney U test corrected with Benjamini and Hochberg method for FDR).

**Figure S3. Dual-strain combinations representative of commensalism, competition, and amensalism imaged after crystal violet staining**

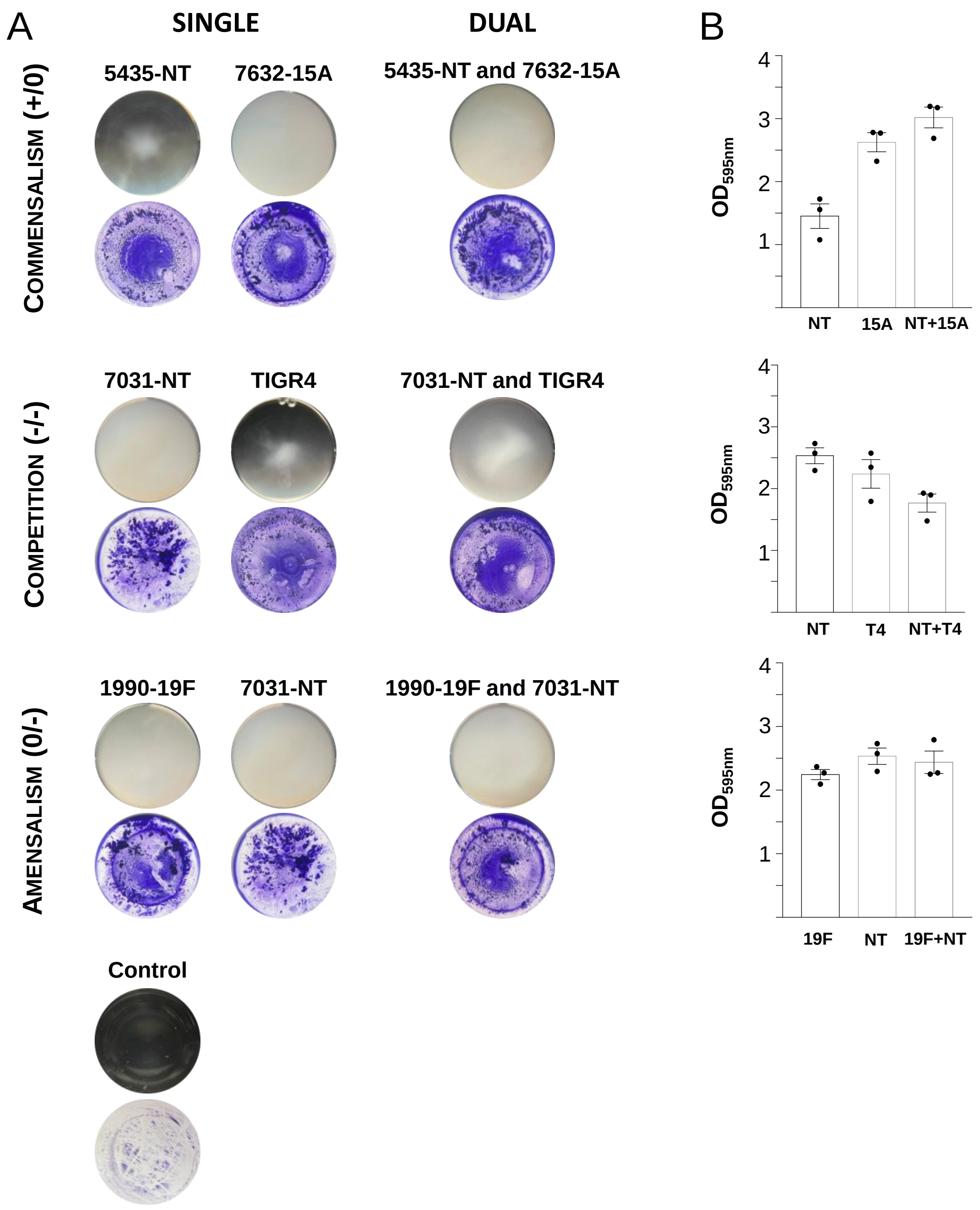


**FIGURE S3. Dual-strain combinations representative of commensalism, competition, and amensalism imaged after crystal violet staining.** Single- and dual-strain biofilms were grown for 72h as described. At 72h, supernatants were removed and biofilms were imaged before and after crystal violet staining **(A)**. For staining, 50uL of crystal violet (CV, 1% w/v) were added to each well and incubated at room temprature for 30 min. CV was removed by inversion and plates were allowed to air-dry for 10 min. Biofilms were washed twice with 500 uL of PBS. Biofilms were imaged on a Zeiss Axio Zoom.V16 sterio microscope equipped with a Zeiss Axiocam 503 mono CCD camera and controlled with the Zen 2.1 software (blue edition), using the 1X 0.25NA objective and bright field optics. Representative images are shown. Wells where only medium was added were used as controls. To estimate the biofilm biomass, CV was solubilized by addition of 1mL of 95% ethanol and incubation with agitation until no stained culture was observed at the bottom of the well. Absorbance at 595nm was measured in a Tecan Infinite F200 plate reader. Absorbance was measured for three biofilms per strain/combination of strains **(B)**.

**Figure S4. Dual-strain combinations representative of commensalism (A), competition (B), and amensalism (C) when grown in mCDM supplemented with 4µg/mL of choline chloride.**

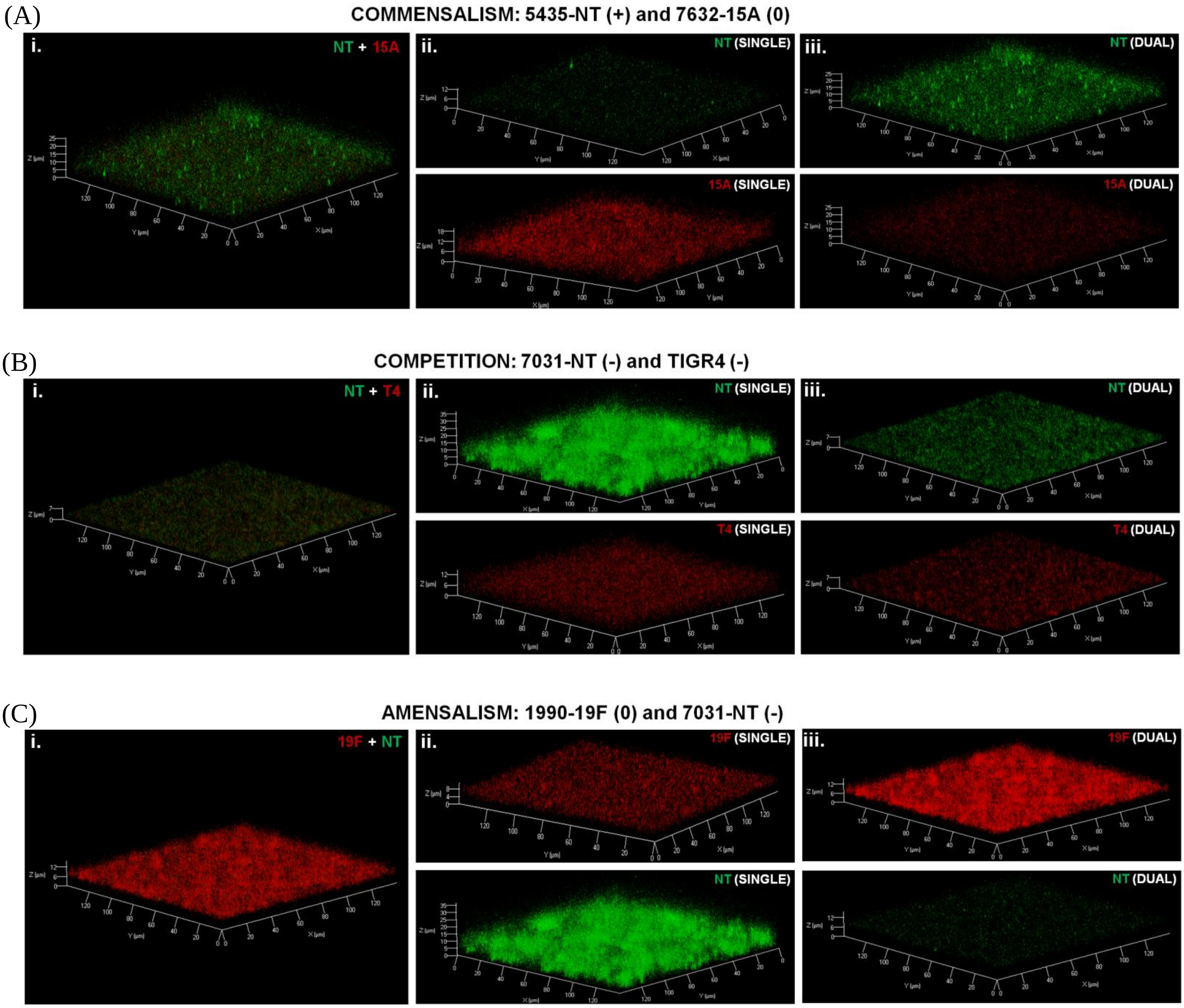


**Figure S4. Dual-strain combinations representative of commensalism (A), competition (B), and amensalism (C) when grown in mCDM supplemented with 4µg/mL of choline chloride.** Strains were tested in dual-strain biofilms (one GFP- and one RFP-labelled strain) in a 1:1 ratio. Reverse labelling combinations were also tested as controls. For comparative analysis, single-strain biofilms were grown in parallel. **(i.) Imaging of dual-strain biofilms. (ii.) Imaging of single strain biofilms. (iii.) Single-channel images of dual-strain biofilms.** All CLSM images are transparent projections of 3D reconstructions from z-sections acquired at 0.81 µm intervals on a Zeiss LSM 880 point scanning confocal microscope using the Airyscan detector, a 20x plan-apochromat 0.8 NA objective (Zeiss) and the 488 nm and 561 nm laser lines.

**Figure S5. Dual-strain combinations representative of commensalism (A), competition (B), and amensalism (C) when grown in mCDM supplemented with 20mg/mL of choline chloride.**

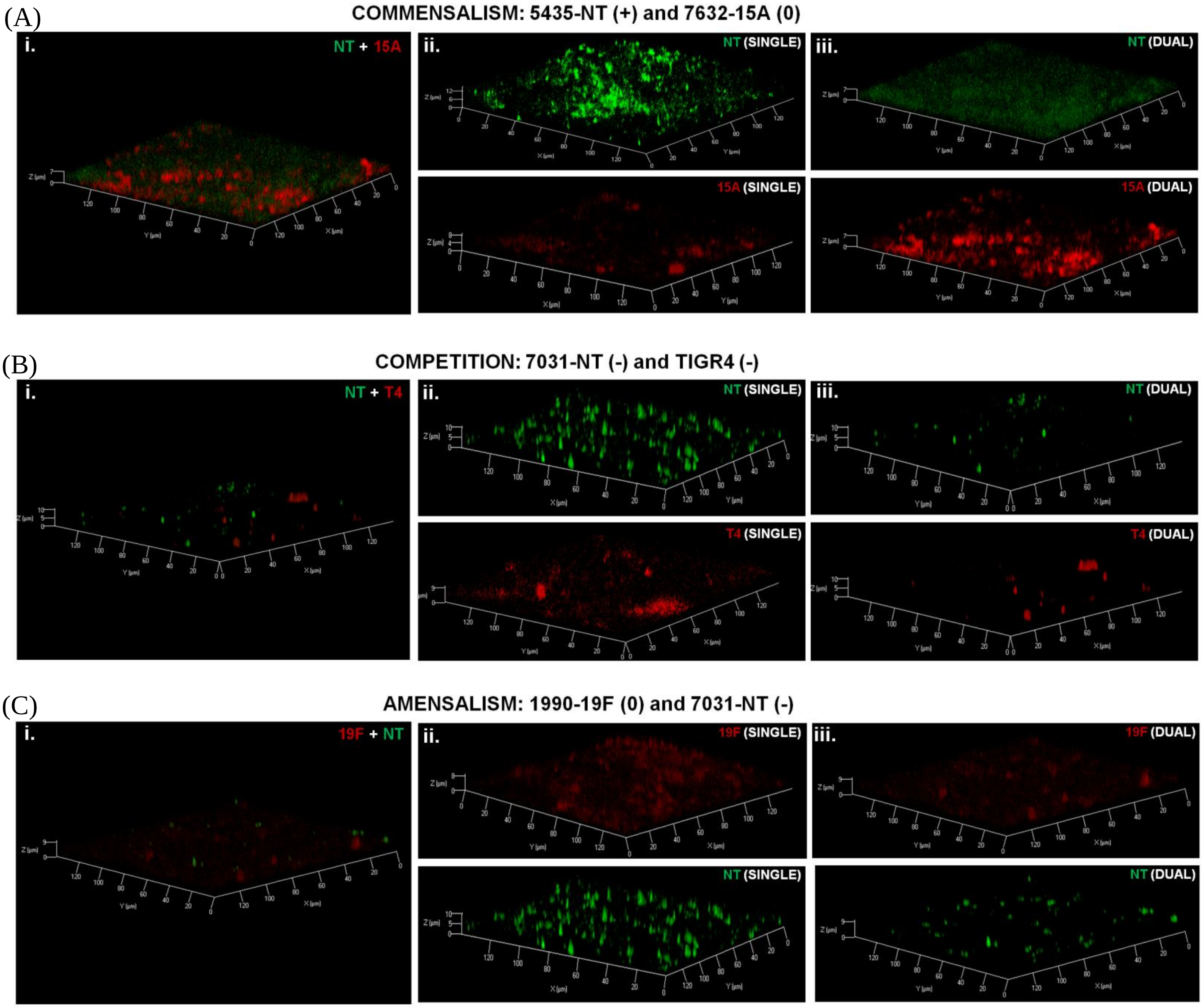


**Figure S5. Dual-strain combinations representative of commensalism (A), competition (B), and amensalism (C) when grown in mCDM supplemented with 20mg/mL of choline chloride.** Strains were tested in dual-strain biofilms (one GFP- and one RFP-labelled strain) in a 1:1 ratio. Reverse labelling combinations were also tested as controls. For comparative analysis, single-strain biofilms were grown in parallel. **(i.) Imaging of dual-strain biofilms. (ii.) Imaging of single strain biofilms. (iii.) Single-channel images of dual-strain biofilms.** All CLSM images are transparent projections of 3D reconstructions from z-sections acquired at 0.81 µm intervals on a Zeiss LSM 880 point scanning confocal microscope using the Airyscan detector, a 20x plan-apochromat 0.8 NA objective (Zeiss) and the 488 nm and 561 nm laser lines.
